# Predictive and prognostic values of serum C1q/tumor necrosis factor-related protein 9 for first-ever ischemic stroke

**DOI:** 10.3389/fneur.2025.1526853

**Published:** 2025-03-07

**Authors:** Yan-Qing Zhang, Hai-Feng Zhang, Xiao-Gang Liu, Rong Li

**Affiliations:** ^1^Department of Anesthesiology, University Town Hospital of Chongqing Medical University, Chongqing, China; ^2^Department of Anesthesiology, The First Hospital, Shanxi Medical University, Taiyuan, China; ^3^Department of Teaching and Experiment Center, Air Force Military Medical University, Xi’an, China; ^4^The Key Laboratory of Biomedical Information Engineering of Ministry of Education of China, School of Life Science and Technology, Xi’an Jiaotong University, Xi'an, China; ^5^Department of Geriatrics, Xijing Hospital, The Fourth Military Medical University, Xi’an, China

**Keywords:** ischemic stroke, CTRP9, adiponectin, prognosis, mortality

## Abstract

**Background:**

The C1q/Tumor Necrosis Factor-related Protein 9 (CTRP9) is a relatively novel adipokine having showed protection on cerebrovascular system. However, its clinical values have not been well established. This work is to evaluate CTRP9 as predictors of onset risk and outcome of ischemic stroke.

**Methods:**

One thousand one hundred and twenty-three patients undergoing first-ever ischemic stroke and 835 controls were enrolled. Serum CTRP9 was determined within 24 h after the onset. One thousand and twenty-six patients were successfully followed up for all-cause and cardiovascular deaths. Stepwise regression was conducted to screen the independent factors of stroke onset in the whole sample and mortality in the patient subgroup. Survival curves were plotted to evaluate the effect of baseline serum CTRP9 on 3-year all-cause and cardiovascular mortalities of stroke patients.

**Results:**

At baseline, prevalence of first-ever onset of ischemic stroke in high CTRP9 group was significantly lower than that in low CTRP9 group (*p* < 0.05) in non-hyperlipidemic subjects. Accumulative all-cause and cardiovascular mortality of patients with high baseline CTRP9 was significantly lower for the first year post stroke onset (*p* < 0.05). Baseline low CTRP9 was one of the independent risk factors of 3-year all-cause mortality (*p* < 0.05) of ischemic stroke patients.

**Conclusion:**

High serum CTRP9 exerted protection against first-ever onset of ischemic stroke in non-hyperlipidemic subjects, and also protected general stroke patients against all-cause and cardiovascular mortality at least 1 year post stroke onset. Our findings in this study may pinpoint both the predictive and prognostic values of CTRP9 as a promising biomarker.

## 1 Introduction

Ischemic stroke, also known as cerebral infarction, refers to localized ischemic softening or necrosis of brain parenchyma caused by cerebral circulation disorders, secondary ischemia, and hypoxic pathological changes (1). Stroke represents the second leading cause of death in the world only after ischemic heart disease, and is the leading cause of disability in adults (2). Ischemic stroke accounts for about 87% of all strokes (3). Therefore, it is important to identify high-risk individuals in the elders especially those already with some predisposing factors.

It has been well-established that adipose tissue has active secretory functions (4). It secretes various adipokines involved in many pathophysiological processes, such as inflammation, energy metabolism, apoptosis, and aging (4–6). Among the adipokines, leptin, adiponectin, and the C1q/Tumor Necrosis Factor-related Proteins (CTRPs) typically play active roles in energy metabolism regulation, vasomotion modulation, platelet activation, and inflammation reaction (7–9). Substantial evidence supports CTRP9 as a beneficial molecule against obesity-related cardiovascular diseases and glycolipid disorders (7, 10). However, the clinical significance of CTRP9 in the onset risk and prognosis of ischemic stroke has not been well explored. Identification of new biomarkers would facilitate early or ultra-early recognition of high-risk patients of ischemic stroke or unfavorable clinical events, and thus introduce better management of patients. Discovery of protective adipokines against unfavorable outcomes of stroke patients may provide novel target and pathway for novel interventions.

## 2 Methods

### 2.1 Study design and approval

This study consisted of both a cross-sectional and a prospective cohort design. The cross-sectional component aimed to assess the relationship between baseline serum CTRP9 levels and the risk of first-ever ischemic stroke, while the prospective cohort component followed stroke patients for up to 3 years to evaluate the association between CTRP9 and long-term mortality. For the cross-sectional study, 1,123 patients with ischemic stroke and 835 control subjects were recruited from departments of geriatrics of Xijing Hospital (Xi’an, China) from January 2016 to November 2019. As of the cohort study, the inception cohort included all patients who met the inclusion criteria at the time of their first-ever ischemic stroke. To avoid potential confounding effect and to control heterogeneity of the study sample, we excluded subjects with any of the following conditions: (1) presence or history of cerebral hemorrhage without pre-existing ischemic stroke; (2) history of ischemic encephalopathy of other causes; (3) previous or current diagnosis of coronary heart disease or coronary syndrome. Any study participant who failed to be followed up was also excluded from the whole study. We also excluded the patients from the whole study who underwent thrombolytic therapy after the stroke.

The study was approved by the Institutional Review Boards of Xijing Hospital (approval number: XJ20150125). All study participants gave written consent for study participation. The study protocol conforms to the ethical guidelines of the 1975 Declaration of Helsinki.

### 2.2 Clinical and laboratory examination

All patients met the diagnostic criteria of the Guidelines for the Prevention of Stroke in Patients with Stroke and Transient Ischemic Attack (American Heart Association and American Stroke Association, 2014). Infarction or absence of ischemic infarct lesion was confirmed by CT or MRI.

About 4 mL of peripheral vein blood was collected immediately after confirmation of ischemic stroke, or at 6:00 a.m. the morning after admission. All blood samples were drawn within 24 h after stroke onset. The blood was placed into EDTA tube for 2 h at room temperature, and centrifuged for 15 min at 3,000 r/min. The serum supernatant was stored in a labeled cryopreservation tube at −80°C freezer. Patient records were reviewed against the including and excluding criteria after patient discharge to determine the participation qualification. Patient height and weight data were out of records because most patients were bedridden.

Serum CTRP9 was measured by use of double antibody sandwich ELISA kits (Shanghai Xitang, China). Each blood sample was measured twice and the results were averaged for subsequent analyses. All the intra-assay and inter-assay coefficients of variation of measures were below 5%.

### 2.3 Follow-up

On entry of the study, all study subjects and their sons or daughters consented to be contacted for the purpose of follow-up. Long-term mortality information was primarily obtained through two ways: first, we established WeChat group in which all patients’ authorized contacts can report any events to the researchers in a real-time manner; second, the researchers call the patients and their children every 3 months from discharge of the hospital for query of survival status. Actually, a substantial proportion of deaths occurred in the hospital, in which case the mortality information was reliable. If out-of-hospital death was reported, the researchers would visit the patients’ contacts in person to make it sure. If the visit was refused, the patients would be excluded from the analyses for mortality.

Cardiovascular mortality is defined as death due to any cardiovascular conditions including ischemic stroke itself, medical implications of stroke, hemorrhagic transformation, recurrent ischemic stroke, new hemorrhagic stroke, myocardial infarction, other cardiovascular condition, and non-neurologic bleeding.

### 2.4 Statistical analyses

Data management and statistical analyses were achieved using Excel and SAS (ver. 9.4, SAS Institute Inc., Cary, NC). Quantitative observations were presented as mean ± SD. Difference between groups were tested by Student’s *t* test or *t’* test. Counting data are conveyed by rate (%) and subjected to chi-square test. Independent factors of risk to stroke onset were determined by use of stepwise logistic regression. *p* values less than 0.05 were considered significant.

Survival curves were estimated by use of the Kaplan–Meier method. Low-and high-CTRP9 statuses were defined as serum CTRP9 below and above the median in the whole study sample, respectively. For low-and high-CTRP9 groups, the survival curves represented the expected proportion of survival. The stepwise regression based on Cox proportional hazard model was conducted to screen the independent factors of 1-year all-cause and cardiovascular mortalities.

## 3 Results

Table 1 shows the general characteristics of the study subjects. There was no significant difference in serum CTRP9 between the stroke and control groups (*p* > 0.05). Age, male gender, family history, smoking, hypertension, diabetes, atrial fibrillation, metabolic syndrome, systolic blood pressure, diastolic blood pressure, homocysteine, and blood sugar were all greater in the stroke group compared to control (*p* < 0.05). The concentration of folic acid, high-density lipoprotein (HDL), low-density lipoprotein (LDL), total cholesterol, and triglyceride, as well as the ratio of hypercholesterolemia and hyperlipidemia were decreased in the stroke group compared to control (*p* < 0.05). Serum CTRP9 was significantly lower (*p* < 0.0001) in the hyperlipidemic group (0.55 ± 0.44) compared to the non-hyperlipidemic group (0.70 ± 0.49).

**Table 1:** Characteristics of the study subjects by ischemic stroke.

Characteristic	Ischemic stroke		*p* value	OR (95% CI)
	Yes (*n* = 1,026)	No (*n* = 835)		
Age	59.97 ± 14.84	51.38 ± 16.92	**<0.0001**	1.035 (1.028–1.041)
Male gender	720 (70.18%)	501 (60.00%)	**<0.0001**	1.569 (1.294–1.902)
Family history	237 (23.10%)	97 (11.62%)	**<0.0001**	2.885 (2.226–3.739)
Smoking	268 (26.12%)	111 (13.29%)	**<0.0001**	2.306 (1.808–2.942)
Hypertension	693 (67.54%)	295 (35.33%)	**<0.0001**	3.809 (3.141–4.620)
Diabetes	239 (23.29%)	93 (11.14%)	**<0.0001**	2.423 (1.869–3.141)
Atrial fibrillation	68 (6.63%)	19 (2.28%)	**<0.0001**	3.048 (1.818–5.113)
Metabolic syndrome	397 (38.69%)	256 (30.66%)	**<0.0001**	1.832 (1.488–2.256)
SBP	145.76 ± 26.48	125.32 ± 17.84	**<0.0001**	1.043 (1.038–1.049)
DBP	83.49 ± 15.16	76.45 ± 10.61	**<0.0001**	1.043 (1.035–1.052)
Folic acid	13.14 ± 10.70	14.86 ± 11.01	**<0.0001**	1.018 (1.009–1.027)
Vitamin B12	354.25 ± 319.38	500.79 ± 428.90	0.7308	1.000 (1.000–1.000)
Homocysteine	16.20 ± 11.73	14.76 ± 14.60	**<0.0001**	1.038 (1.028–1.049)
HDL	1.09 ± 0.28	1.20 ± 0.31	**<0.0001**	0.368 (0.300–0.451)
LDL	2.16 ± 0.84	2.36 ± 0.76	**<0.0001**	0.692 (0.633–0.756)
Total cholesterol	3.77 ± 1.07	4.27 ± 1.04	**<0.0001**	0.730 (0.688–0.775)
Triglyceride	1.47 ± 0.79	1.67 ± 1.06	**<0.0001**	0.710 (0.645–0.781)
Hyperlipidemia	248 (24.17%)	301 (36.05%)	**0.0001**	0.665 (0.540–0.819)
Hypercholesterolemia	9 (0.88%)	22 (2.63%)	**0.0137**	0.374 (0.171–0.818)
Blood sugar	6.09 ± 2.42	5.03 ± 1.20	**<0.0001**	1.483 (1.377–1.596)
CTRP9	0.66 ± 0.51	0.66 ± 0.48	0.4691	0.993 (0.825–1.194)

OR, odds ratio; CI, confidence interval; SBP, systolic blood pressure; DBP, diastolic blood pressure; HDL, high-density lipoprotein; LDL, low-density lipoprotein; CTRP9, C1q/TNF-related protein 9. Significant *p* values are in bold.

First, we compared serum CTRP9 between the stroke and control groups for potential significant predictors of stroke onset. In the whole sample, serum CTRP9 did not show significant difference between stroke patients and controls (Table 1). In the subgroup analyses with respect to whether being hyperlipidemic, there was significant difference in serum CTRP9 (*p* = 0.007) between patients and controls in the non-hyperlipidemic subgroup. Driven by this finding, we afterwards focused on the non-hyperlipidemic subgroup and conducted stepwise logistic regression to screen the independent factors of stroke risk. All available baseline characteristics listed in Table 1, except hyperlipidemic status and its directly related variables including high-density lipoprotein, low-density lipoprotein, total cholesterol, triglyceride, and hypercholesterolemia, were adopted in the initial regression model. In the fitted model, the significant independent risk factors of stroke included age, smoking, stroke family history, systolic blood pressure, blood sugar, and homocysteine. Among all the assessed baseline characteristics, CTRP9 is the unique independent protective factor of onset of ischemic stroke (Table 2).

**Table 2:** Independent factors of ischemic stroke in the non-hyperlipidemia subgroup.

Characteristic	Ischemic stroke		*p* value	OR (95% CI)
	Yes (*n* = 566)	No (*n* = 457)		
Age	61.78 ± 14.77	52.58 ± 17.22	<0.0001	1.047 (1.035–1.060)
Smoking	185 (32.69%)	61 (13.35%)	0.005	1.958 (1.224–3.130)
Stroke family history	155 (27.39%)	56 (12.25%)	<0.0001	2.689 (1.663–4.347)
SBP	143.43 ± 23.94	124.34 ± 16.28	<0.0001	1.043 (1.032–1.053)
CTRP9	0.65 ± 0.48	0.76 ± 0.49	0.0008	0.657 (0.512–0.841)
Blood sugar	5.62 ± 2.08	4.88 ± 1.22	<0.0001	1.490 (1.282–1.731)
Homocysteine	16.81 ± 13.21	14.48 ± 10.89	0.0467	1.015 (1.000–1.030)

OR, odds ratio; CI, confidence interval; SBP, systolic blood pressure; CTRP9, C1q/TNF-related protein 9; For age, SBP, CTRP9, blood sugar, and homocysteine, OR represents the relative risk of mortality associated with a one-unit increase in these continuous variables.

All variables listed in Table 1, except hyperlipidemia status and its directly related variables including high-density lipoprotein, low-density lipoprotein, total cholesterol, triglyceride, and hypercholesterolemia, were included in the initial model to screen the significant independent factors of stroke onset in the non-hyperlipidemia subgroup by use of stepwise logistic regression. This table lists the significant independent factors in the final logistic model.

We failed to reach 97 stroke patients in the follow-up. The dropout rate was 8.61%. The remaining 1,026 patients were successfully followed up for 34.1 months on average. The median of serum CTRP9 in the whole study sample was 0.53 μg/mL, which was used to define low-and high-CTRP9 statuses in the study sample. Causes of death by CTRP9 status in the follow-up among the stroke patients is presented in Table 3. As shown, ischemic stroke itself, medical implications of stroke, and hemorrhagic transformation represented the dominant causes of death.

**Table 3:** Causes of death among stroke patients in the follow-up period by CTRP9 status.

Cause of death	Number of deaths (% to *n*)	
	Low CTRP9	High CTRP9
Ischemic stroke itself	25	26
Medical implications of stroke	20	3
Hemorrhagic transformation	26	23
Recurrent ischemic stroke	4	13
New hemorrhagic stroke	1	3
Myocardial infarction	7	10
Other cardiovascular condition	11	8
Non-neurologic bleeding	5	3
Nonvascular condition	20	1
Undetermined	18	36
Total deaths	137	126

CTRP9, C1q/TNF-related protein 9. Low CTRP9/high CTRP9, serum CTRP9 is lower/higher than the median of CTRP9 concentration in the whole sample.

**Figure 1:**
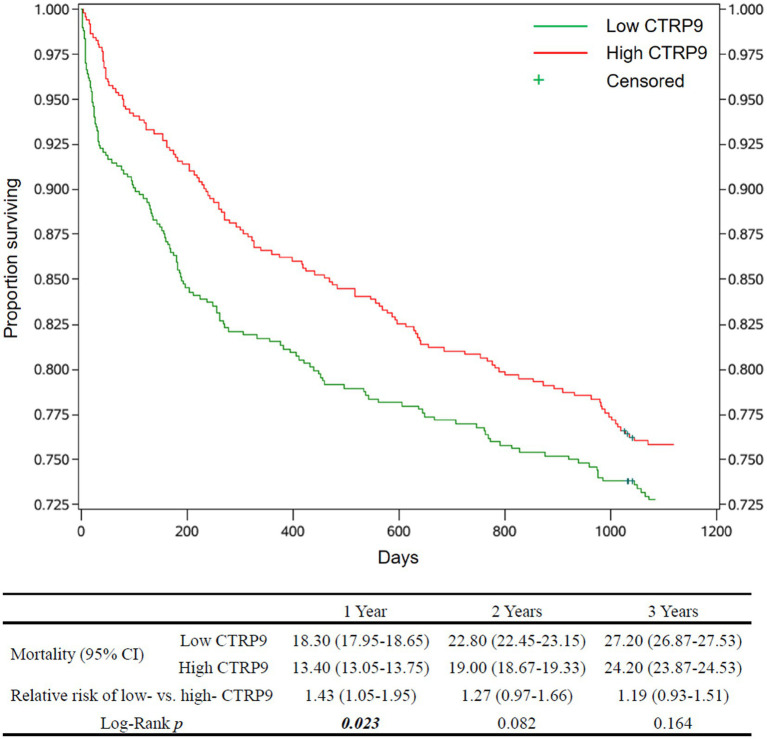
Survival curves and all-cause mortality of the patients by CTRP9 level. Cumulative survival curves of the Low-CTRP9 and High-CTRP9 groups are shown in the upper figure. The table below shows estimated mortalities of the two groups, the relative death risk of low- versus high- CTRP9 group (high CTRP9 group as reference), and the Log-rank *p*-values for statistically testing the equality of all-cause mortality between the two groups. The values in in brackets are 95% confidence intervals. The significant *p* values (*p* < 0.05) are in italic bold. CTRP9, C1q/TNF-related protein 9; Low CTRP9/ high CTRP9, serum CTRP9 is lower/higher than the median of CTRP9 concentration in the whole sample.

**Figure 2:**
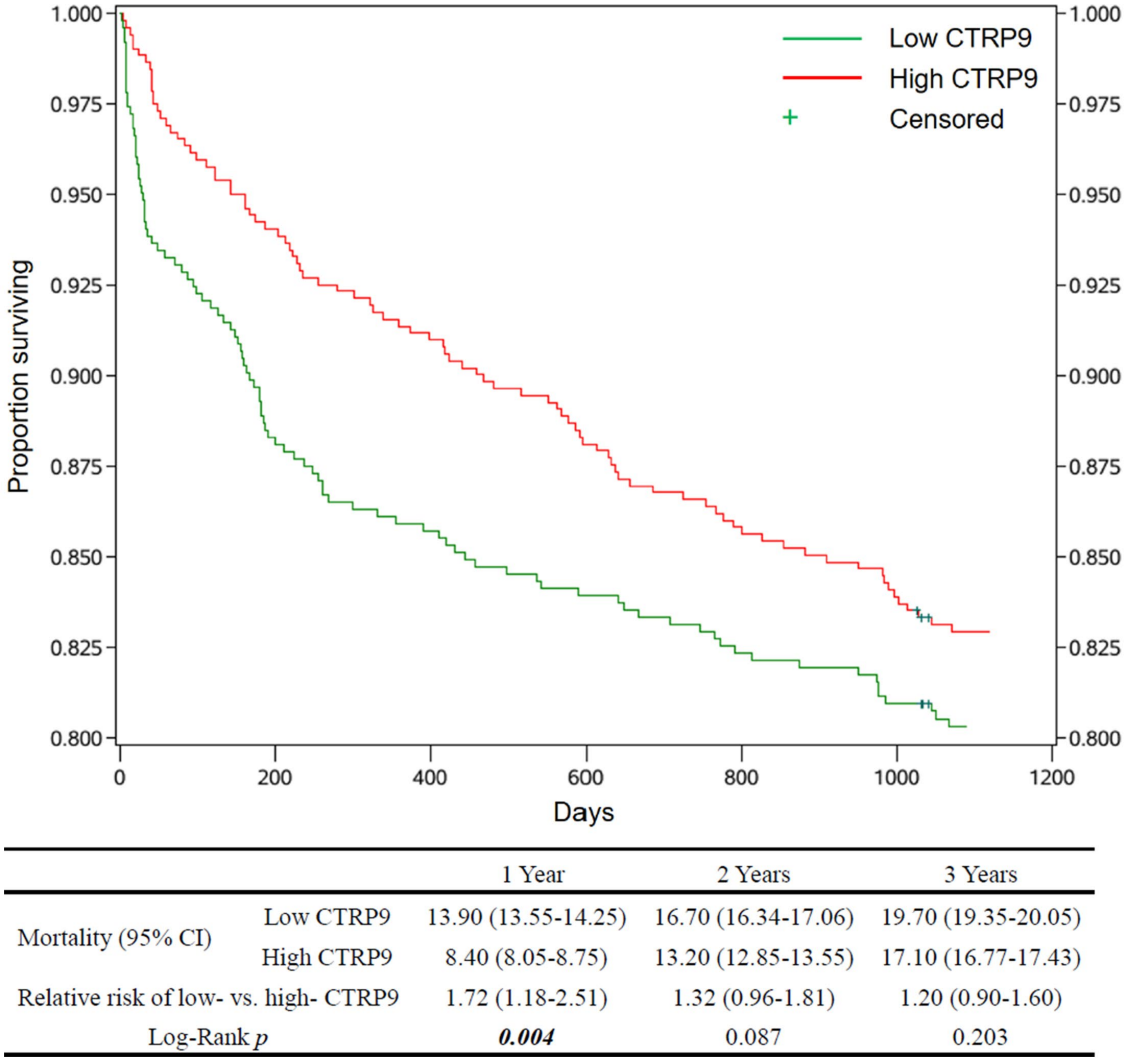
Survival curves and cardiovascular mortality of the patients by CTRP9 level. Cumulative survival curves of the Low-CTRP9 and High-CTRP9 groups are shown in the upper figure. The table below shows mortalities of the two groups, the relative death risk of Low- versus High-CTRP9 (high CTRP9 group as reference), and the Log-rank *p*-values for statistically testing the equality of cardiovascular mortality between the two groups. The values in brackets are 95% confidence intervals. The significant *p* values (*p* < 0.05) are in italic bold. CTRP9, C1q/TNF-related protein 9; Low CTRP9/ high CTRP9, serum CTRP9 is lower/higher than the median of CTRP9 concentration in the whole sample.

The estimates of cumulative survival, relative risk of death, and survival curves, as well as the *p*-values for testing the equality of mortality between the low-and high-CTRP9 groups, are shown in Figure 1 for all-cause mortality and Figure 2 for cardiovascular mortality. There was clear trends that low CTRP9 increased mortality compared with the high-CTRP9 group, especially the difference reached statistically significant (*p* = 0.023 for all-cause mortality and *p* = 0.004 for cardiovascular mortality, respectively) for the first year after stroke onset.

The applied medications of the patients included aspirin, renin angiotensin system inhibitors, beta blockers, and lipid lowering medications. There is no significant difference of applied medications between the patients of low-and high-CTRP9 groups (details not shown).

**Figure 3:**
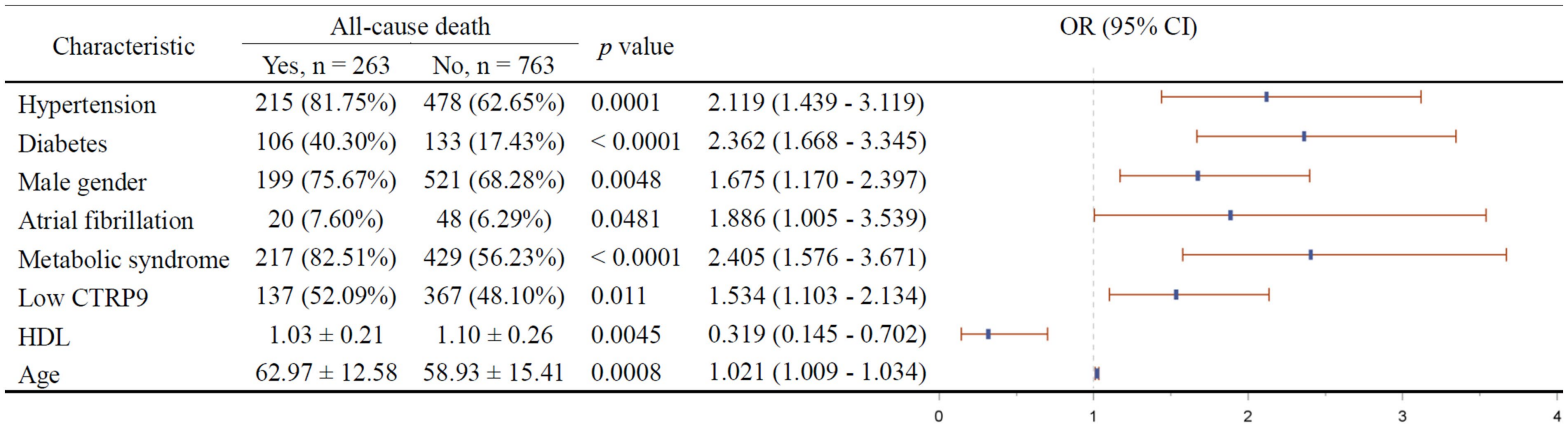
Independent factors of 1-year all-cause mortality of stroke patients. In the right figure, X axis represents odds ratio (OR). Y axis represents the corresponding independent factors. OR < 1 means the corresponding factor is a protective factor, while OR > 1 meaning a risk factor. The vertical dashed line is placed where OR = 1. The vertical lines in blue represent the estimates of OR, with the extending horizontal bars representing their 95% confidence intervals. CTRP9, C1q/TNF-related protein 9; HDL, high-density lipoprotein; Low CTRP9, low level of CTRP9 defined by less than median of serum CTRP9 in the whole sample.

**Figure 4:**
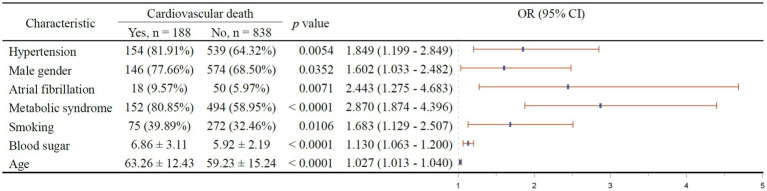
Independent factors of 1-year cardiovascular mortality of stroke patients. In the right figure, X axis represents odds ratio (OR). Y axis represents the corresponding independent factors. OR < 1 means the corresponding factor is a protective factor, while OR > 1 meaning a risk factor. The vertical dashed line is placed where OR = 1. The vertical lines in blue represent the estimates of OR, with the extending horizontal bars representing their 95% confidence intervals.

The results of multivariable stepwise cox regression intended to screen for independent factors of 1-year all-cause mortality is shown in Figure 3. It turned out that hypertension (*p* = 0.0001), diabetes (*p* < 0.0001), male gender (*p* = 0.0048), atrial fibrillation (*p* = 0.0481), metabolic syndrome (*p* < 0.0001), low CTRP9 (*p* = 0.011), and advanced age (*p* = 0.0008) are among the independent risk factors of all-cause mortality, while only high HDL (*p* = 0.0045) was indicative of low all-cause mortality. As shown in Figure 4, we did not identify any independent protective factor against 1-year cardiovascular mortality. Hypertension (*p* = 0.0054), male gender (*p* = 0.0352), atrial fibrillation (*p* = 0.0071), metabolic syndrome (*p* < 0.0001), smoking (*p* = 0.0106), high blood sugar (*p* < 0.0001), and advanced age (*p* < 0.0001) imposed higher risk of cardiovascular mortality.

## 4 Discussion

Our main finding in this study is that elevated CTRP9 level was not only an independently protective factor against onset of ischemic stroke in non-hyperlipidemic subjects, but also consistently was indicative of lower all-cause and cardiovascular mortality in general patients of first-ever ischemic stroke for at least 1 year. The results of stepwise cox regression further implies that serum CTRP9 may functionally protect stroke patients against all-cause mortality.

The body’s inflammatory response has significant bearing in development of ischemic stroke (11). Ischemic stroke shares common pathological mechanisms with cardiovascular diseases such as coronary heart disease (12). Clinical trials demonstrate increased CTRP9 is an independent protective factor for coronary heart disease, with correlation to coronary artery disease degree (13). To exclusively determine the role of serum CTRP9 in pathophysiology of ischemic stroke, we excluded coronary heart disease patients from this study.

CTRP9, a novel adipokine first reported in 2009, expresses predominantly in adipose tissue (14). CTRP9 regulates energy metabolism, modulates vasomotion, protects endothelial cells, inhibits platelet activation and pathological vascular remodeling, stabilizes atherosclerotic plaques, and protects the heart (15).

Emerging evidence suggests that CTRP9 may exert neuroprotective effects upon diverse central nervous system diseases (16). CTRP9 has a high affinity to APN receptor 1, which is widely expressed in the central nervous system, especially in neurons (17, 18). APN receptor 1 agonists protect the brain against ischemic stroke and intracerebral hemorrhage, via inhibition of neuronal apoptosis (19, 20). Nevertheless, few research reports the association of serum CTRP9 level with ischemic stroke risk in human beings (21). Recently, Yang et al. investigated the association between serum CTRP9 levels and ischemic stroke prognosis in a cohort study with a one-year follow-up. In line with our findings, their study demonstrated that CTRP9, along with its lipid ratios, was associated with stroke severity and unfavorable outcomes. In contrast, our study provides novel insights by focusing on a larger cohort with a longer follow-up period (3 years) (22). More specifically, they found that CTRP9/lipids ratios provided stronger association with ischemic stroke than CTRP9 alone, which is consistent with our findings that high serum CTRP9 only exerted protection against first-ever onset of ischemic stroke in non-hyperlipidemic subjects. These studies consistently imply hyperlipidemia may impart important influence on the association of CTRP9 with ischemic stroke, suggesting the value of calibration of serum CTRP9 with blood lipids to provide more precise prediction.

Closely related to abnormal adipose metabolism, hypercholesterolemia is an important and independent risk factor for ischemic stroke (1, 3). As expected, serum CTRP9 in the hyperlipidemic group were decreased compared to the non-hyperlipidemic group. To avoid the confounding variable of lipid concentration, a subgroup analyses per serum lipid level was completed in our study. In the non-hyperlipidemic group, serum CTRP9 was significantly lower in stroke patients compared to control. This implies that decreased levels of CTRP9 may be risk factors of ischemic stroke independent of hypercholesterolemia. Meanwhile, high levels of serum CTRP9 are an independent protective factor for metabolic syndrome, correlating with decreased hyperlipidemia indicators such as cholesterol, triglyceride, and LDL (23). Binary logistic regression in the non-hyperlipidemic group revealed age > 60, smoking, hypertension, and low vitamin B12 were risk factors of ischemic stroke, while increased CTRP9 was a protective factor of ischemic stroke. In the case that CTRP9 was eliminated from the initial model, high concentration of HMW exerted protective factor for ischemic stroke (data not shown).

Interestingly, although high CTRP9 exerted protection against onset of stroke only in the non-hyperlipidemic subgroup, we also observed its protective effect against mortality in the non-hyperlipidemic subgroup and in hyperlipidemic subgroup, besides the foregoing detailed results of the survival analyses and cox regression in the whole sample. These findings may highlight the importance of adopting hyperlipidemia status as covariate in clinical studies on adipokines.

CTRP9 is a homologous substance of APN, and also have beneficial metabolic regulatory and vasoprotective effects. In this study, we found that high level of baseline serum CTRP9 exerted significant protection against all-cause and cardiovascular mortality in patients of ischemic stroke. Validation of this finding might provide important tool to identify those high-risk stroke patients earlier and thus improve management of patients.

The major limitation of this study is that the first step of the study is a cross-sectional study, making it difficult to establish causal inference of high CTRP9 as a risk factor of onset of ischemic stroke. We tried the best to measure serum CTRP9 as soon as possible after onset of stroke to eliminate the possibility of change in serum CTRP9 brought by stroke onset. Blood of all the patients were drawn within 24 h after stroke onset. The average time from onset to blooding drawing is 9.1 h. Furthermore, it’s not likely for onset of stroke to largely influence serum CTRP9 from the viewpoint of biological rationale. Specifically, (1) CTRP9 is mostly secreted by adipose tissue; (2) There is no report on the concentration of CTRP9 in brain; however, as a homolog of CTRP9, brain concentration of APN is about 1/1,000 the serum concentration largely attributable to the existence of blood–brain barrier. In addition, a substantial body of literature focused on the potential effects of serum CTRP9 on risk to onset of ischemic stroke. These facts could serve to strengthen the causal inference supporting our finding that high CTRP9 is a protective factor of onset of ischemic stroke in non-hyperlipidemic subjects. Another limitation might be that we did not stratify patients based on metabolic status, such as obesity and diabetes, which may influence CTRP9 expression. Yang et al. highlighted that CTRP9 levels could vary significantly in different subtypes of ischemic stroke and in patients with cardiovascular comorbidities (22). Future research should explore whether CTRP9’s predictive value remains consistent across diverse patient populations, particularly in those with metabolic syndrome. Additionally, while we focused on all-cause and cardiovascular mortality, further investigations into CTRP9’s mechanistic pathways in stroke recovery and secondary prevention are warranted.

## 5 Conclusion

In conclusion, we propose that increased serum CTRP9 levels are associated with a lower risk of ischemic stroke onset and reduced long-term mortality. CTRP9 may serve as a potential biomarker for both stroke risk assessment and prognosis. Further studies are warranted to confirm these findings.

## Data Availability

The raw data supporting the conclusions of this article will be made available by the authors, without undue reservation.
